# Social-Demographic Indicators, Cognitive Ability, Personality Traits, and Region as Independent Predictors of Income: Findings from the UK Household Longitudinal Study (UKHLS)

**DOI:** 10.3390/jintelligence6020019

**Published:** 2018-03-26

**Authors:** Adrian Furnham, Helen Cheng

**Affiliations:** 1 Department of Leadership and Organizational Behavior, Norwegian Business School, Nydalsveien 37, 0484 Oslo, Norway; 2 ESRC Centre for Learning and Life Chances in Knowledge Economies and Societies, Institute of Education, University of London, London WC1H 0AL, UK; h.cheng@ucl.ac.uk

**Keywords:** net monthly income, cognitive ability, big-five personality factors, education and occupation, Government Office Region, Longitudinal

## Abstract

This paper reports on a longitudinal study of over 12,000 people based on the UK Household Longitudinal Study data. We were interested in their monthly income (as the criterion variable) as it related to their gender, age, education, occupation, personality, intelligence, and region where they lived (as the predictor variables). Correlations showed that, after occupation and education, gender and cognitive ability (particularly numeric ability) were the strongest correlates of income. Hierarchical regressions showed that age and gender accounted for 9% of the variance, intelligence and personality added another 5%, and education and occupation added a further 15%, while region added a further 1%. All four models were statistically significant (*p* < 0.001). The study suggests that, in future research of this kind on the personal correlates of income, social-demographic, psychological, and regional factors all need to be considered. Limitations are acknowledged.

## 1. Introduction

What factors determine a person’s annual income? Is it primarily a function of their demography (age, sex, region where they live) or their psychological make-up (intelligence and personality)? If so, how do these factors contribute to their level of education and specific occupation which are always closely related to annual salary? Also what part does geographic location play. This longitudinal study uniquely combines demographic data (sex, age, education and occupation), individual difference data (personality and intelligence) as well as geographic region to exam the correlates of salary (monthly income).

Personal wealth as a research topic has drawn interest from economists, educationists, psychologists, and sociologists [1]. The different disciplines emphasize different factors that are associated with the financial status of people in adulthood. Previous studies have established findings on the links between family background, childhood intelligence, and later educational and occupational outcomes [2,3,4,5,6,7,8,9,10,11,12,13,14,15]. There are obvious associations between demographic variables and income: males tend to obtain higher salaries than females, older people are paid more than younger people, and those from richer regions (i.e., capital cities) are paid more than those from poorer regions (i.e., countryside or deprived regions). Further, it is self-evident that brighter people do well in educational settings, which leads to higher-paid professional jobs.

There is also a literature on the relationship between personality, occupational choice, and financial success. Studies suggest that conscientiousness is positively, and neuroticism negatively, related to career success and income [16,17,18]. Duckworth and Weir [19] found that one standard deviation (SD) of conscientiousness raised average earnings by $1500 (which was about five percent of the mean of $30,000), while the same (i.e., 1 SD) for emotional stability (low neuroticism) increased earnings by $700. Nabeshima and Seay [20], however, found extraversion and conscientiousness positively, and agreeableness negatively, associated with net worth. They hypothesized that it was the risk-taking nature of extraverts and the disciplined, organized, and practical nature of conscientious people that explained these results. Equally they thought it was the empathy, unselfishness, and generosity of highly agreeable people that led to their lower net worth. The personality variables added around 2.5 of the explained variance after sex, age, ethnicity, marital status, education, and household income.

Although there are slightly different results from studies looking at personality trait correlates of income, the three that are implicated most often are conscientiousness, (low) neuroticism, and agreeableness, in that order. These three make up a higher order factor labelled by Digman [21] as ‘Alpha’, while the other two (extraversion and openness) were called ‘Beta’. Digman also described factors of ‘communion’ and ‘agency’, where agency refers to strivings for mastery, power, self-assertion, and self-expansion, and communion refers to the urge toward community and the relinquishing of individuality. In this study, we combined the Big Five into the two factors to test whether Alpha was much more positively related to income than Beta.

Other individual difference correlates of income studies have looked at factors like mental toughness, defined as commitment, challenge, control, and confidence [22], and core self-evaluations [23].

Furnham and Cheng [7] investigated a longitudinal data set of 4790 adults examining a set of socio-demographic and psychological factors that influence adult financial well-being. They showed that childhood intelligence, locus of control, education, and occupation were all independent predictors of adult financial well-being for both men and women. Parental social status and psychological distress were also significant predictors of the outcome variable for men, but not for women. For women, compared to men, the effects of current occupation and childhood intelligence on the outcome variable appeared to be stronger. The strongest predictor of adult financial well-being was current occupational prestige.

In another study, Furnham and Cheng [8] examined a sample comprised of 4537 British adults in a longitudinal study, which was primarily interested in their salary/wage earning measured at age 54. Correlational results showed that parental social class, childhood cognitive ability, traits extraversion, emotional stability, conscientiousness, openness, education, and occupation, as well as gender, were all significantly associated with adult earning ability. Multiple regression analysis showed that childhood cognitive ability, the traits of conscientiousness and openness, educational qualifications, and occupational prestige were significant and independent predictors of adult earning ability accounting for 30% of the total variance.

This study set out to replicate some of the above findings using different measures of cognitive ability tests and personality factors. In addition, it adds to what is already known because it explored the effects of a set of socio-economic and psychological factors on net income taking account of the effect of geographical region, using data collected from a large representative population sample in the UK, the UK Household Longitudinal Study (UKHLS). We also had the advantage in this study of examining specific intelligence test scores to determine if numerical rather than verbal intelligence was a better predictor of income. This, we believe, is another unique feature of this study. Moreover, we examined personality in terms of the higher order Alpha and Beta factors, which is novel in this research area.

It is self-evident that salaries for similar jobs differ considerably according to the wealth of the region, yet few, if any, psychological studies have ever considered this. Region is often considered as an explanatory variable in economic and sociological studies of income and other outcomes such as health, but rarely in psychology studies.

Based on previous studies we derived four hypotheses:

(H1) Cognitive ability would be a significant correlate of income. (H2) Alpha Factor (the higher order of personality factor combining agreeableness, conscientiousness, and reversed neuroticism scores) would be a significant correlate of income. (H3) There would be a significant gender effect on the outcome variable, with females earning less than males. (H4) Regional effects would be modest but significant after taking account of the effects of all social-demographic and psychological factors on the outcome variable.

## 2. Method

The UK Household Longitudinal Study (UKHLS) named Understanding Society is an innovative world-leading study following the lives of 40,000 UK households to provide valuable evidence about 21st century UK life and how it is changing [24]. It captures (every year starting in 2009 with the Wave 5 data available in November 2015) important information about people’s social and economic circumstances, attitudes, behaviours, and health. The study is longitudinal in its design and of high quality. Data in Wave 1 (2009), Wave 3 (2011), and Wave 5 (2013) were used in the study. These data were collected each year for the same participants from Wave 1 (2009) to Wave 5 (2013); age, gender, and government region were measured in Wave 1 (2009); cognitive assessment and personality traits were measured in Wave 3 (2011); current occupation and the outcome variable of income was measured in Wave 5 (2013).

### 2.1. Participants

The study was based on a sample of 12,138 participants (males = 5247 and females = 6891) with an age range from 16 to 82 (mean = 39.3, SD = 11.9). In all, 5.8% were <20 years old; 17.2% between 20 and 29; 26.1% between 30 and 39; 29.4% between 40 and 49%; 18.0% between 50 and 59; and 3.5% 60 years old and over. We had information on net monthly income and current occupation in Wave 5, a set of cognitive tests, the Big Five personality factors, and educational qualifications obtained (all in Wave 3). Information on the Government Office Region in the UK was also available.

### 2.2. Measures

1 *Cognitive Ability Tests*

A set of four sets of cognitive ability tests were used in the study: 1. *Immediate Word Recall* (number of correct items). For this task, the computer reads a list of 10 words to standardize the presentation and speed of the word list. The interviewer checks if the respondent can hear the computer playing a short test message. If the voice cannot be heard, the interviewer checks again following adjustment of the volume. If the respondent still cannot hear the computer’s voice, the interviewer reads the words at a slow steady rate of about one word every two seconds. The list of words is not repeated. No aids are allowed for the test. 2. *Subtract* (number of correct answers). In this test, the respondent is asked to give the correct answer to a series of subtraction questions. Starting at 100, the interviewer asks the respondent to subtract 7. At the next question, the respondent is asked to subtract 7 again, and so on. There is a sequence of five subtractions. No materials or aids were allowed for this test. 3. *Verbal fluency* (count of correct answers). In this test, participants were asked to name as many animals as they could in one minute. 4. *Numeric Ability* (count of items answered correctly). In this task, respondents were asked up to five questions that are graded in complexity. The standardized cognitive ability tests scores were combined and converted into British IQ scores with mean = 100 and SD = 15 in the following regression analysis.

2 *Personality Factors*

Personality traits are classified according to the ‘Big Five’ taxonomy: agreeableness (A), conscientiousness (C), extraversion (E), neuroticism (N), and openness (O). Three items for each factor were available in Wave 3 with inevitably modest alphas. These five factors were computed into two higher order factors named ‘Alpha Factor’ (A + C + N) and ‘Beta Factor’ (E + O) because of the low alphas of the individual factors. Cronbach’s alpha was 0.65 for Alpha Factor and 0.65 Beta Factor.

3 *Education*

Educational qualifications were ranged from 0 = no qualification to 5 = university degree in Wave 3.

4 *Occupation*

Current occupation (in Wave 5) was measured by the Registrar General’s measure of social class (RGSC). RGSC is defined according to occupational status and lifestyle [25]. It was coded on a 6-point scale: I: unskilled (3.9%); II: partly skilled (16.5%); IIIM: skilled manual (11.4%); IIIN: skilled non-manual (23.7%); V: managerial/technical (38.2%); VI: professional (6.3%) [26].

5 *Region*

The Government Office Region includes 12 regions in the UK: London, North East, North West, Yorkshire, and the Humber, East Midlands, West Midlands, East of England, South East, South West, Wales, Scotland, and Northern Ireland. A dummy variable was created using London as a reference region.

6 Income

In Wave 5, participants were asked about their current net payment per month. Incomes were logged in the following analyses.

## 3. Results

### 3.1. Correlational Analysis

Correlation analysis was conducted to examine the associations between earning ability and a set of psychological and socio-demographic variables in the study. Results are shown in Table 1.

Table 1 shows that, among the variables examined in the study, cognitive ability tests scores (immediate word recall, subtract, verbal fluency, and numeric ability), the personality trait Alpha Factor (but not Beta Factor), education, and occupation were all significantly (*p* < 0.001) and positively associated with income. There was also a gender effect on the outcome variable; men had a significantly higher income then women (*p* < 0.001).

Table 1 also shows that cognitive ability tests scores were significantly associated with education and occupation in the expected direction. There is always the concern that education, occupation, and income are so highly intercorrelated that there is a serious co-linearity problem. However, as can be seen from Table 1, none of the three correlations was *r* > 0.50, and the correlation between education and income was only *r* = 0.33.

Cognitive ability tests immediate word recall and verbal fluency, numeric ability were significantly (*p* < 0.001) and positively correlated with Beta Factor; and to a lesser extent, immediate word recall was negatively and numeric ability was positively correlated with Alpha Factor (*p* < 0.05).

Table 1 illustrates two important issues with respect to intelligence test scores. First, whereas three of the four (word recall, subtract, and verbal fluency) were nearly all identically correlated with income (*r* = 0.12; *r* = 0.13), the correlation with numerical ability was more than twice as high (*r* = 0.29). Next, the correlation between the four intelligence scores was modest (0.14 < *r* > 0.33), indicating some measure of ‘g’.

### 3.2. Regression Analysis

Following this, a hierarchical regression analysis was carried out using log income as the dependent variable. Table 2 shows the results.

Table 2 shows that in Step 1 (Model 1), both gender and age were significant predictors of income, accounting for 9% of variance. In Step 2 (Model 2), after a set of psychological variables were entered into the equation, it was shown that cognitive ability and Alpha Factor were significant predictors of income, which in addition accounted for 5% variance. Gender and age remained as significant predictors of the outcome variable. In Step 3 (Model 3), after the two social variables were entered, it was shown that education and occupation both were significant predictors of income, accounting for a further 15% variance, and gender, age, cognitive ability, and Alpha Factor remained significant. In Step 4 (Model 4), it was shown that, compared with the London region, all other 11 regions were significantly and negatively associated with the outcome variable, indicating that participants in these 11 regions had lower incomes than participants in the London region. Although the effects of region on income was modest (1% additional variance), the model was significant (*F* (11, 10,320) = 225.25, *p* < 0.001). In the final model, cognitive ability, Alpha Factor, gender, and age remained as significant predictors of the outcome variable, accounting for, in total, 30% of the variance.

## 4. Discussion

This study replicated the results of various others using different measures of intelligence and personality and different populations in terms of size and nationality. In accordance with many other studies, the strongest correlates of monthly (and annual) salary and accumulated wealth are education and occupation, which are usually closely related [7,8]. In this study, educational qualifications and occupational status were correlated *r* = 0.47 which is similar to many other studies. Highly paid, high status jobs usually require extensive educational qualifications.

The results of this study also highlighted the importance of intelligence, particularly numerical intelligence. As may be expected, the correlations between all four intelligence measures and education were highly significant and in the range 0.15 < *r* < 0.34 and very similarly to the correlations between intelligence and occupation 0.13 < *r* < 0.31. Again, this is a well-established finding that intelligence predicts educational and occupational outcomes, which in turn are correlated with income [4].

What is particularly interesting about the results of this study was the relationship between numerical ability and income. Whilst few dispute the evidence for general intelligence (g), it is particularly interesting to compare the results for numerical and verbal intelligence (correlated at 0.30). The correlation between numerical intelligence and income was over twice that of verbal ability (*r* = 0.29 vs. *r* = 0.13). Whilst both related abilities are clearly important in the workplace, it seems that jobs associated with high numerical abilities (banking, consulting, engineering, and finance) are most financially rewarded. Many studies have shown how general mental ability, in combination with other factors such as confidence or self-beliefs, are related to occupational attainment, yet few have looked at relationships between intelligence, personality, and success together [27,28].

There was also a large effect for gender, showing that females earn much less than males [29]. There are many explanations for this fact, and many attempts have been made to reduce the wage gap [30]. However, the size of the correlation and the beta suggest that gender remains a very important factor in income.

This study also considered the role of personality factors in income, which has been explored over the past decade [1]. Whilst there have inevitably been inconsistencies in the results, many study have identified conscientiousness and neuroticism as the two most powerful and predictive correlates of occupational success measured by many criteria including income. In this study, we examined the relationship between Digman’s higher order factors, i.e., Alpha and Beta Factors. We found the former rather than the latter related to income.

The regression results showed that, of the two demographic factors, gender was much more powerful than age in explaining income, and together these two variables accounted for around a tenth of the variance. Further, results showed that the two psychological variables, namely personality and intelligence, accounted for an additional 5% variance, with intelligence being a stronger predictor of income than personality. However, once education and occupation were taken into account, the effect of intelligence on income decreased and was about the same as personality. This, in part, may be due to the amount of covariance among these variables.

The regressions showed an additional factor unique to this study and rarely considered in psychological studies of this sort. Both economists and sociologists tend to emphasize structural, and geographers regional, factors in (part) explaining the distribution of wealth. In this study, we were able to take this factor into consideration when examining the wealth of individuals as the data set had 12 regional classifications: Eight classifications for England, Scotland, Wales, and Northern Ireland, as well as London. Yet the data showed this added only 1% of the variance once the other factors had been taken into consideration. This was a surprising and unexpected finding, as it is well established that there are often big income differences between those in rural vs urban areas. This may be due to a sampling problem where the households selected are essentially unrepresentative of the overall distribution of a specific region. Certainly this finding needs replication.

### Limitations

The sample with complete data had a slight under-representation of lower/manual occupational classes and had slightly higher IQ scores (IQs of 104 in the present study and IQs of 100 in the total sample of the UKHLS), which may provide a small bias in these results (i.e., the findings were more conservative). Moreover, we did not include other variables, such as ethnicity, parental occupation, and health. In early work, we did look at these but missing data and group size preclude us from including them in the final analysis. Furthermore, we had data on where people lived rather than where they worked. It is likely that some of the participants were commuters in the sense that they lived outside, but worked in, London.

This study does suggest some interesting future research in the area. For instance, we were unable to examine the effect of any family instability in a household on subsequent earnings. An unstable home could affect education, job choices, and risk-taking tendencies, which could have a very different impact on males and females.

## Figures and Tables

**Table 1:** Pearson correlations matrix between income, a set of cognitive ability tests, personality factors, education, and occupation.

	Variables	Mean SD					Correlation						
			1	2	3	4	5	6	7	8	9	10	11
1.	Income (log)	3.09 (0.31)	_										
2.	Gender	0.57 (0.50)	−0.29 ***	**_**									
3.	Age	39.29 (11.88)	0.05 ***	0.02	_								
4.	Immediate word recall	6.73 (1.49)	0.12 ***	0.05 ***	−0.14 ***	_							
5.	Subtract	4.18 (1.52)	0.13 ***	−0.09 ***	−0.01	0.14 ***	_						
6.	Verbal fluency	23.30 (6.65)	0.13 ***	−0.02	−0.01	0.31 ***	0.15 ***	_					
7.	Numeric ability	3.77 (1.02)	0.29 ***	−0.21 ***	0.05 ***	0.27 ***	0.34 ***	0.30 ***	_				
8.	Alpha Factor	46.45 (6.71)	0.06 ***	0.01	0.18 ***	−0.03 *	−0.01	0.02	0.03 *	_			
9.	Beta Factor	27.75 (5.72)	0.02	0.02	−0.08 ***	0.09 ***	−0.01	0.11 ***	0.05 ***	0.31 ***	_		
10.	Educational qualifications	3.26 (1.48)	0.33 ***	0.02	−0.15 ***	0.27 ***	0.15 ***	0.21 ***	0.34 ***	−0.03 *	0.12 ***	_	
11.	Current occupation	3.91 (1.32)	0.41 ***	0.01	−0.01	0.21 ***	0.13 ***	0.21 ***	0.31 ***	0.02	0.08 ***	0.47 ***	_

*Note:* * *p* < 0.05; *** *p* < 0.001. Standard deviations (SD) are given in parentheses. Variables were scored such that a higher score indicated being female, a higher score on current earnings, higher cognitive ability test scores, higher cores on personality factors, highest educational qualification, and a more professional occupation for participants. Correlation analysis was weighted with UK sampling weight.

**Table 2:** Predicting adult income from gender and age, cognitive ability, personality factors, education and occupation, as well as region.

Measures	Model 1		Model 2		Model 3		Model 4		
	Beta	*t*	Beta	*t*	Beta	*t*	Beta	*t*	*p #*
Gender	−0.30 ***	30.44	−0.27 ***	28.69	−0.30 ***	34.22	−0.30 ***	34.22	<0.001
Age	0.06 ***	6.22	0.06 ***	5.72	0.07 ***	7.66	0.07 ***	7.39	<0.001
Cognitive ability			0.21 ***	22.42	0.06 ***	6.26	0.07 ***	7.11	<0.001
Personality trait Alpha Factor			0.05 ***	5.16	0.06 ***	6.54	0.07 ***	7.02	<0.001
Personality trait Beta Factor			−0.01	0.10	−0.02	1.90	−0.02	1.91	0.080
Educational qualifications					0.18 ***	17.87	0.17 ***	16.90	<0.001
Current occupation					0.30 ***	30.65	0.30 ***	30.17	<0.001
Government office region *(London as the reference region)*									
North East							−0.06 ***	6.21	<0.001
North West							−0.08 ***	6.92	<0.001
Yorkshire and the Humber							−0.09 ***	7.73	<0.001
East Midlands							−0.08 ***	7.50	<0.001
West Midlands							−0.06 ***	5.45	<0.001
East of England							−0.05 ***	4.63	<0.001
South East							−0.06 ***	5.30	<0.001
South West							−0.10 ***	8.90	<0.001
Wales							−0.06 ***	6.03	<0.001
Scotland							−0.08 ***	7.02	<0.001
Northern Ireland							−0.05 ***	5.09	<0.001
*Variance explained*	*R*^2^ *adjusted = 0.09* F (2, 10,336) = 479.21 ***		*R*^2^ *adjusted = 0.14* F (3, 10,333) = 208.36 ***		*R*^2^ *adjusted = 0.29* F (2, 10,331) = 555.03 ***		*R*^2^ *adjusted = 0.30* F (11, 10,320) = 225.25 ***		

*** *p* < 0.001. # Significance in the final model. Regression analysis was weighted with UK sampling weight.
